# Recent Drug Overdose Mortality Decline Compared With Pre–COVID-19 Trend

**DOI:** 10.1001/jamanetworkopen.2024.58090

**Published:** 2025-02-05

**Authors:** Mathew V. Kiang, Keith Humphreys

**Affiliations:** 1 Department of Epidemiology and Population Health, Stanford University School of Medicine, Stanford, California; 2 Department of Psychiatry and Behavioral Sciences, Stanford University School of Medicine, Stanford, California; 3 Center for Innovation to Implementation, Veterans Affairs Palo Alto Health Care System, Palo Alto, California

## Abstract

This cross-sectional study compares annual state-level fatal drug overdose rates from 2020 through 2023 with the expected fatal drug overdose rates based on trajectories before the COVID-19 pandemic.

## Introduction

The 14.5% year-over-year decrease in fatal drug overdoses in the 12 months preceding June 2024^1^ has raised hopes that the deadly dynamics of the crisis have fundamentally shifted. This speculation would be more plausible if the decline occurred across the US and was due to more than returning to prepandemic levels of growth.^2^ To evaluate the geographic distribution of the recent improvement in drug deaths and determine whether it is driven by more than the waning exacerbating outcomes of the COVID-19 pandemic, we compare annual state-level fatal drug overdose rates from 2020 through 2023 with the expected fatal drug overdose rates based on their pre-2020 trajectories.

## Methods

This cross-sectional study followed the Strengthening the Reporting of Observational Studies in Epidemiology (STROBE) reporting guideline. The Stanford University institutional review board deemed this study exempt from review because data were deidentified and publicly available.

We obtained annual age-standardized fatal drug poisoning data from the CDC WONDER database for 48 states and the District of Columbia (hereafter, referred to as states) from 1999 to 2023. Rates for North Dakota and South Dakota were not available due to insufficient sample size. To estimate the pre-COVID-19 trajectory, we fit separate joinpoint regression models to each state using data from 1999 to 2019. The final joinpoint model was selected using a permutation test.^3^ We then projected the final joinpoint model from 2020 to 2023 and compared the observed rates with this counterfactual trajectory of drug-related mortality. Observed rates were considered higher or lower than expected if they were outside the 95% CI of the expected rates. Statistical significance was set at *P* < .05. Data were accessed on September 24, 2024, and analyzed from September 24 to November 24, 2024, using R version 4.4.1 (R Foundation). Additional information is included in the eMethods in Supplement 1.

## Results

**Figure 1.**
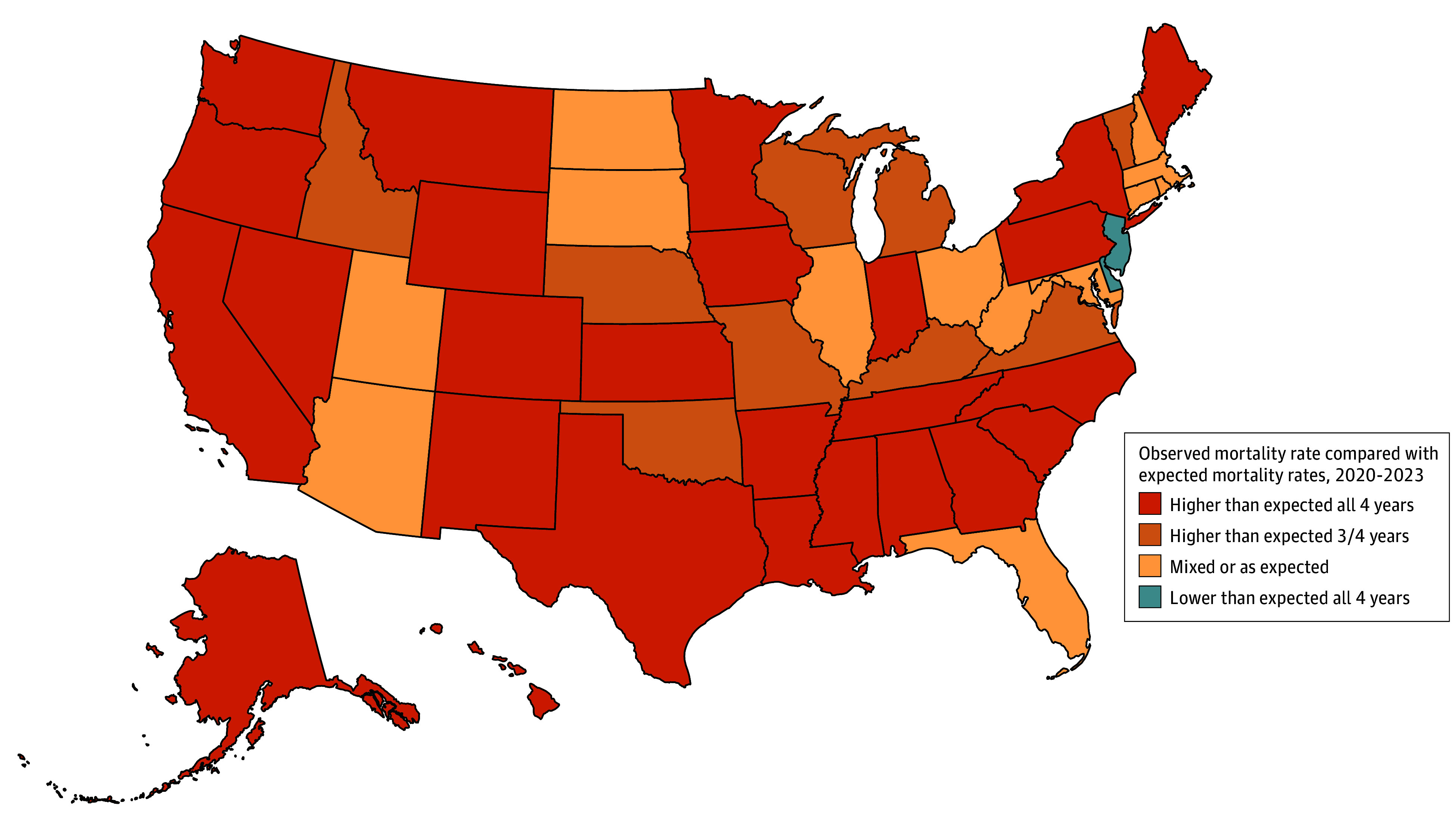
Summary of the Observed Mortality Rate Compared With the Expected Mortality Rate for 2020 Through 2023 for Each State Expected mortality is based on a joinpoint regression model fit to data from 1999 to 2019 and estimated for 2020 to 2023.

**Figure 2.**
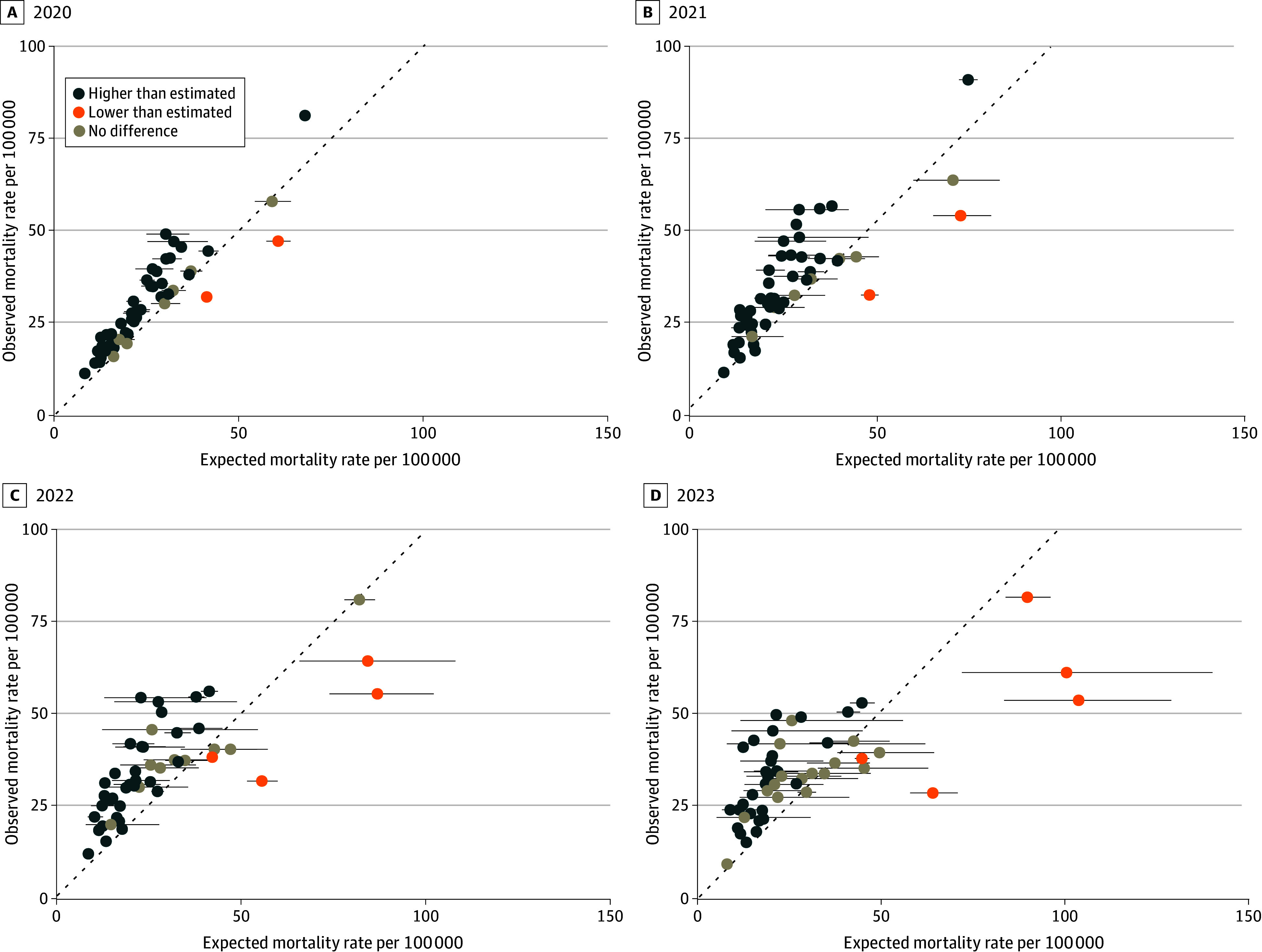
Observed vs Estimated Drug-Related Mortality Rates by State, 2020 to 2023 The dotted line indicates where the observed and expected mortality rates are equal, and the dots indicate observations that were statistically higher (blue) or lower (orange) and not statistically different (gray).

This study included data from 49 states. When comparing the observed drug-related mortality rates in 2020 through 2023 with their pre-2020 trajectories, most states had higher than expected mortality for all 4 years (26 of 49 states [53%]) or 3 of 4 years (9 of 49 states [18%]) (Figure 1 and Figure 2). In the 4 years between 2020 and 2023, 46 states (94%) had higher drug-related mortality than their 2019 rates. Only New Jersey (in 2022 and 2023), Delaware (in 2020), and New Hampshire (in 2020) were exceptions. Nine states (18%), mostly west of the Mississippi, experienced rapidly increasing mortality relative to their pre-2020 trajectory (California, Colorado, Oregon, Washington, Alaska, Oklahoma, Texas, Wyoming, and Alabama). Conversely, 4 states (8.2%) experienced a spike in drug-related mortality early in the COVD-19 pandemic, and rates appeared to be consistently decreasing toward or below their pre-2020 trajectory after the pandemic (Arizona, Tennessee, Louisiana, and Florida).

## Discussion

Drug overdose deaths have increased exponentially since 1979.^2^ This rate of increase accelerated during the COVID-19 pandemic^4^ but has since waned. When comparing recent drug-related mortality rates with their pre-2020 trajectory, the vast majority of states remained higher than expected. In the 4 years between 2020 and 2023, nearly all states had higher drug-related mortality rates than their 2019 rates. Only New Jersey (in 2022 and 2023), Delaware (in 2020), and New Hampshire (in 2020) were exceptions.

Study limitations include potential differences in reporting over time and the use of provisional death data in recent years. Additionally, several states are experiencing increasing drug-related mortality relative to their pre-2020 trajectories. These states are generally west of the Mississippi River and may reflect a shifting illicit drug supply^5^ as new areas become saturated with illicitly manufactured synthetic opioids.^6^ The fourth wave of deaths from methamphetamine and the increasing presence of xylazine and nitazines in illicit drug markets have the potential to reverse the progress made in the past 12 months.

The recent decrease in overdose deaths is welcome news, but celebrating or relaxing would be premature. The decline in mortality may be largely due to the waning of the exacerbating impact of the COVID-19 pandemic. Furthermore, in the western US, overdoses may increase rather than decrease in the next few years.

## Notes

Supplement 1.
eMethods.

Supplement 2.
Data Sharing Statement
