# Supplementary material for: Recent Drug Overdose Mortality Decline Compared With Pre–COVID-19 Trend
Source: JAMA Netw Open. 2025 Feb 5;8(2):e2458090. doi: 10.1001/jamanetworkopen.2024.58090 (PMC11800014; doi:10.1001/jamanetworkopen.2024.58090)
Supplement: Supplement 1. — eMethods. [file jamanetwopen-e2458090-s001.pdf]

Supplemental Online Content

Kiang MV, Humphreys K. Recent drug overdose mortality decline compared with pre-COVID trend. *JAMA Netw Open*. 2025;8(2):e2458090. doi:10.1001/jamanetworkopen.2024.58090

eMethods.

This supplemental material has been provided by the authors to give readers additional information about their work.

© 2025 Kiang MV et al. *JAMA Network Open*.

eMethods

We used state-specific, age-standardized drug-related mortality rates downloaded from the CDC WONDER database on September 24, 2024. Data from 1999 through 2020 came from the final multiple cause of death files. Data from 2021 on came from the provisional multiple cause of death files. North Dakota and South Dakota were excluded from analysis due to small sample sizes and suppressed data.

We then fit joinpoint regression models for each state using data from 1999 through 2019. The optimal joinpoint model was selected using the permutation test with 4,499 permutations. Sensitivity analyses suggest our models are robust to alternative methods for model selection. Statistical significance was defined at the .05 level. Using the final joinpoint model, we predicted mortality rates for 2020 through 2023 and the corresponding 95% confidence interval of these predictions. We defined observed rates that fall outside of the 95% confidence interval of the prediction to be "higher" or "lower" than expected. We also conducted an omnibus paired samples, Wilcoxon signed-rank test to non-parametrically assess if observed rates were different from predicted rates overall in 2020 through 2023. Results of the signed-rank test were highly significant ($p < .00001$), indicating observed rates were overall higher than predicted rates.

Reproducible code and data are available online at https://github.com/mkiang/drug_mortality_2023. Joinpoint regression models were estimated using the NCI Joinpoint Regression Program (available at https://surveillance.cancer.gov/joinpoint/).

© 2025 Kiang MV et al. *JAMA Network Open*.
