# Supplementary material for: Recent Drug Overdose Mortality Decline Compared With Pre–COVID-19 Trend
Source: JAMA Netw Open. 2025 Feb 5;8(2):e2458090. doi: 10.1001/jamanetworkopen.2024.58090 (PMC11800014; doi:10.1001/jamanetworkopen.2024.58090)
Supplement: Supplement 2. — Data Sharing Statement [file jamanetwopen-e2458090-s002.pdf]

Data Sharing Statement

Data
Data available: Yes
Data types: Other (please specify)
Additional Information: All data are publicly available. Reproducible code and analytic data will be shared upon publication via a Github repository.
How to access data: All data are publicly available. Reproducible code and analytic data will be shared upon publication via a Github repository.
When available: With publication

Supporting Documents
Document types: Statistical/analytic code
How to access documents: All data are publicly available. Reproducible code and analytic data will be shared upon publication via a Github repository.
When available: With publication

Additional Information
Who can access the data: All data are publicly available. Reproducible code and analytic data will be shared upon publication via a Github repository.
Types of analyses: Data can be used for any purpose within the constraints of the NCHS data use agreement.
Mechanisms of data availability: All data are publicly available. Reproducible code and analytic data will be shared upon publication via a Github repository.
